# Establishing community based cohorts: approaches, methods and challenges

**DOI:** 10.1186/1753-6561-7-S5-O4

**Published:** 2013-08-30

**Authors:** S Mehendale

**Affiliations:** 1 National Institute of Epidemiology (NIE), Chennai, India

## 

India with a population of 1.2 billion is changing rapidly with increasing literacy, declining sex ratio, huge urban growth, high population density and a rising proportion of young people. Some communicable diseases show a declining trend while others show an upward trend. The proportion of deaths and burden of disease due to chronic disease risk factors are on the rise.

Resource limited countries like India face several practical problems, the most prominent being the difficulty to convince policy makers that non-communicable diseases are silent killers. The size and diversity of the population necessitates large cohorts resulting in significant cost escalation. In addition infrastructure, manpower and resource limitations preclude the availability of quality data. The operational and management challenges include consistency of data capture across diverse regions, building and sustaining clinical infrastructure, long term data storage and ethical issues. The scientific requirements in undertaking population based cohort studies in India include identification of susceptible individuals, tracking of migrants and qualitative studies to supplement quantitative data. Mechanisms to ensure community participation like need based benefits for the community, proper feedback and networking with non-governmental organizations and private health care providers are also important.

In addition to the information available from routine surveillance, medical records and national programmes, a well planned cohort study can provide incidence rates, predictors of diseases, sub-clinical infections, individual, family and community parameters, in-migration and out-migration, early indications of impending outbreaks, health and treatment seeking behaviour and impact of individual or community level clinical or behavioural interventions. Senior level health managers and experts have identified non-communicable diseases as a public health research priority next only to maternal and child health and it is necessary to initiate population based cohort studies and plan interventions accordingly.

